# Epigenetic Regulation of Gfi1 in Endocrine-Related Cancers: A Role Regulating Tumor Growth

**DOI:** 10.3390/ijms21134687

**Published:** 2020-06-30

**Authors:** Nadia Ashour, Javier C. Angulo, Ana González-Corpas, María J. Orea, María V. T. Lobo, Ramón Colomer, Begoña Colás, Manel Esteller, Santiago Ropero

**Affiliations:** 1 Departamento de Biología de Sistemas, Unidad Docente de Bioquímica y Biología Molecular, Universidad de Alcalá, Alcalá de Henares, 28054 Madrid, Spain; nadiashourfernandez@gmail.com (N.A.); agcorpas@gmail.com (A.G.-C.); oreamariajesus@gmail.com (M.J.O.); begona.colas@uah.es (B.C.); 2 Servicio de Urología, Hospital Universitario de Getafe, Fundación para la Investigación Biomédica del Hospital Universitario de Getafe, Universidad Europea de Madrid, Getafe, 28905 Madrid, Spain; jangulo1964@gmail.com; 3 Departamento de Biomedicina y Biotecnología, Universidad de Alcalá; Instituto Ramón y Cajal de Investigaciones Sanitarias (IRYCIS), 28054 Madrid, Spain; mval.toledo@uah.es; 4 Medical Oncology Department, Instituto De Investigación Sanitaria La Princesa, HU La Princesa, 28029 Madrid, Spain; rcolomer@seom.org; 5 Department of Medicine, Universidad Autónoma de Madrid, 28049 Madrid, Spain; 6 Josep Carreras Leukaemia Research Institute (IJC), 08916 Badalona, Catalonia, Spain; mesteller@carrerasresearch.org; 7 Centro de Investigacion Biomedica en Red Cancer (CIBERONC), 28040 Madrid, Spain; 8 Institucio Catalana de Recerca i Estudis Avançats (ICREA), 08010 Barcelona, Catalonia, Spain; 9 Physiological Sciences Department, School of Medicine and Health Sciences, University of Barcelona (UB), 08028 Barcelona, Catalonia, Spain

**Keywords:** prostate cancer, breast cancer, Gfi1, DNA methylation

## Abstract

Prostate and breast cancer constitute the most common cancers among men and women worldwide. The aging population is one of the main risk factors for prostate and breast cancer development and accumulating studies link aging with epigenetic changes. Growth factor independence-1 (Gfi1) is a transcriptional repressor with an important role in human malignancies, including leukemia, colorectal carcinoma, and lung cancer, but its role in prostate and breast cancer is unknown. We have found that Gfi1 epigenetic silencing is a common event in prostate and breast cancer. Gfi1 re-expression in prostate and breast cancer cell lines displaying Gfi1 epigenetic silencing decreases cell proliferation, reduced colony formation density, and tumor growth in nude mice xenografts. In addition, we found that Gfi1 repress alpha 1-anti-trypsin (AAT) and alpha 1-anti-chymotrypsin (ACT) expression, two genes with important functions in cancer development, suggesting that Gfi1 silencing promotes tumor growth by increasing AAT and ACT expression in our system. Finally, Gfi1 epigenetic silencing could be a promising biomarker for prostate cancer progression because it is associated with shorter disease-free survival. In conclusion, our findings strongly indicate that Gfi1 epigenetic silencing in prostate and breast cancer could be a crucial step in the development of these two-well characterized endocrine related tumors.

## 1. Introduction

Prostate and breast cancer are the commonest endocrine-related cancers worldwide. Breast cancer is the most common cancer among women in western countries, whereas prostate cancer constitutes the most frequent malignancy diagnosed in males in developed countries and the second leading cause of cancer mortality in men. These tumor types share risk factors and etiology. Prostate cancer development depends on the androgen receptor, while breast cancer primarily depends on the estrogen receptors. These transcription factors regulate oncogenic signaling pathways leading tumor initiation and progression, allowing their use as therapeutic targets for these tumor types [1].

Growth factor independence-1 (Gfi1) is a zinc-finger transcriptional repressor with critical functions in various developmental processes, including hematopoietic and non-hematopoietic tissues differentiation such as inner ear hair cells, lung and intestine. Gfi1 was originally identified in an insertional-mutagenesis-based study to identify genes implicated in the progression to IL-2 independent growth of T-cell lymphoma lines [2].

Most of the studies regarding Gfi1 function in normal tissues have been focused on the hematopoietic system. Gfi1 expression is required for commitment and development of B and T-cells in bone marrow and thymus. Lack of Gfi1 expression induces cell death, inhibits cell proliferation, and blocks differentiation in early steps of T lymphopoiesis [3]. Mutations in the gene that codify for Gfi1 are associated with Severe Congenital Neutropenia (SCN) [4]. On the contrary, other studies reveal the role of Gfi1 in restricting the proliferation of hematopoietic stem/progenitor cells [5,6]. Although most of the work about Gfi1 has focused on the hematopoietic system, recent studies indicate that this transcriptional repressor is essential for differentiation and survival of several solid organ cell types by antagonizing the Notch signaling pathway. Gfi1 regulates the differentiation of hair cells of the inner ear [7], and survival of cochlear ganglion neurons and cerebellar Purkinje cells [8]. This gene is also required for neuroendocrine pulmonary differentiation [9] and enteroendocrine differentiation of Globet and Paneth cells of the colon and small intestine [10,11].

Gfi1 is also implicated in cancer development, but the available data are contradictory. Gfi1 gain of function has been found in chronic myeloid leukemia (CML) and acute myeloid leukemia (AML) [12,13] suggesting that Gfi1 is an oncogene in this tumor types. On the contrary, transgenic mice for Gfi1 expression do not show an unusual incidence of leukemia [14,15]. Hock et al. reported that Gfi1 is essential to restrict proliferation of hematopoietic stem cells [5], suggesting that Gfi1 can function as a tumor suppressor gene. Gfi1 loss of expression have been also reported in non-small cell lung carcinoma (NSCLC) and colorectal carcinoma. In NSCLC Gfi1 re-expression induces cell cycle arrest, and the patients showing Gfi1 expression show a trend towards better prognosis and longer survival [16]. In colorectal carcinoma Gfi1 silencing influences metastasis spread [17]. However, little is known about the function of Gfi1 in prostate and breast cancer.

Despite the number of studies showing the loss of Gfi1 expression in cancer, there is no studies about the cellular processes promoting its silencing. Transcriptional inactivation by CpG island promoter hypermethylation is an alternative mechanism for gene inactivation in cancer that has emerged as promising biomarker for cancer diagnosis and prognosis. 

The aging population is the main risk factor for developing prostate and breast cancers, but the factors mediating this phenomenon are not fully known. Epigenetic changes are one of the hallmarks of aging suggesting that age-related epigenetic changes may play a key role in prostate and breast cancer development. DNA methylation profile is linked to estrogen receptor status while androgen receptor activity is highly dependent of the epigenetic modifiers. In a previous study showing the gene hypermethylation profile of prostate cancer, we found gfi1 hypermethylated in 37% of the tumor samples [18]. In this study, we have found that Gfi1 epigenetic silencing is a common event in prostate and breast cancer. In these tumor types, Gfi1 re-expression decreases cell proliferation, reduced colony formation density and tumor growth in nude mice xenografts, supporting the hypothesis of a tumor-suppressor role for Gfi1.

## 2. Results

### 2.1. Gfi1 Is Hypermethylated in Prostate and Breast Cancer Cell Lines

**Figure 1:**
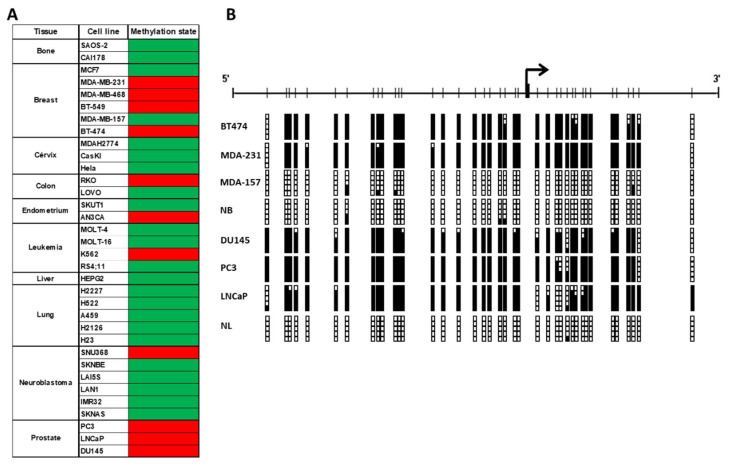
(**A**) Analysis of Gfi1 methylation status in human cancer cell lines. Methylated or unmethylated cell lines are showed by red or green squares respectively. (**B**) Schematic depiction of the Gfi1 promoter region around the corresponding transcription start site (thick black arrows). CpG dinucleotides are represented as short vertical lines. Results of bisulfite genomic sequencing of 5 individual clones are shown. Presence of a methylated or unmethylated cytosine is indicated by a black or white square, respectively.

In a previous study showing a DNA hypermethylation profile for prostate cancer we found Gfi-1 hypermethylated in 37% of the cases and unmethylated in the normal tissues analyzed [18]. Since Gfi1 loss of expression is a common event in different tumor types and its biological role in prostate has not been defined, we decided to characterize the DNA methylation status of Gfi1 promoter in the LNCaP, PC3, and DU145 prostate cancer cell lines by methylation-specific PCR (MSP) and bisulfite genomic sequencing of a region surrounding the transcriptional start site. Gfi1 was hypermethylated in the three prostate cancer cell lines analyzed and was unmethylated in normal lymphocytes (Figure 1B). To test whether Gfi1 hypermethylation is tumor specific or a general event in cancer, we screened a variety of cancer cell lines corresponding to different tumor types (Figure 1A). Interestingly, significant Gfi1 promoter hypermethylation was found in four breast cancer cell lines (MDA-MB-231, MDA-MB-463, BT474 and BT549) and was unmethylated in normal breast indicating that this is not a general event (Figure 1B). Gfi1 was unmethylated in leukemia cell lines as was expected since it has been proposed as an oncogene in this tumor type. Then, we continue the study with prostate and breast cancer cell lines because these are two well characterized endocrine-related cancers sharing great similarities in key hormone signaling pathways controlling tumor growth and differentiation [19].

**Figure 2:**
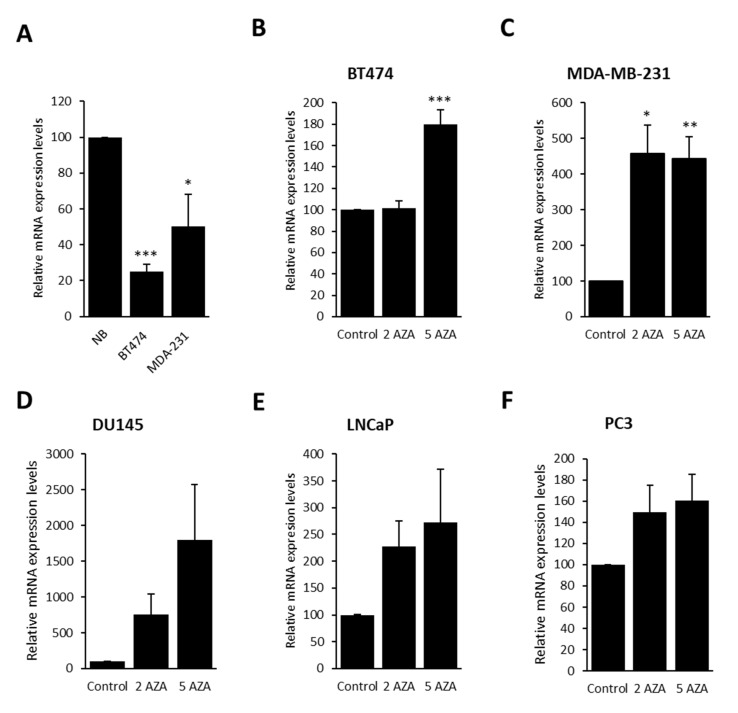
QRT-PCR showing the mRNA expression levels of Gfi1 in breast cancer cell lines (**A**). The treatment with 2 µM (2 AZA) and 5µM (5 AZA) of t1he demethylating agent 5-aza-2-deoxycytidine reactivates Gfi1 expression in methylated cell lines (**B–F**). Data shown represent the mean ± s.e.m. of three independent experiments completed in triplicate. * *p* < 0.05, ** *p* < 0.01, *** *p* < 0.001.

Once Gfi1 promoter hypermethylation was confirmed, we assessed the association of this epigenetic aberration and Gfi1 mRNA expression levels by qRT-PCR. The breast cancer cell lines MDA-MB-231 and BT-474 showing Gfi1 hypermethylated, express very low levels of Gfi1 mRNA compared with normal breast (Figure 2A). The treatment with the demethylating agent 5’-aza-2´-deoxycytidine restores Gfi1 expression in the cell lines, MDA-MB-231, BT-474, DU145, PC3, and LNCaP, hypermethylated at the Gfi1 promoter (Figure 2B–F), establishing a link between Gfi1 promoter hypermethylation and transcriptional silencing.

### 2.2. Gfi1 Re-Expression Genes Decreases Cell and Tumor Growth and Reduces the Expression of Target

**Figure 3:**
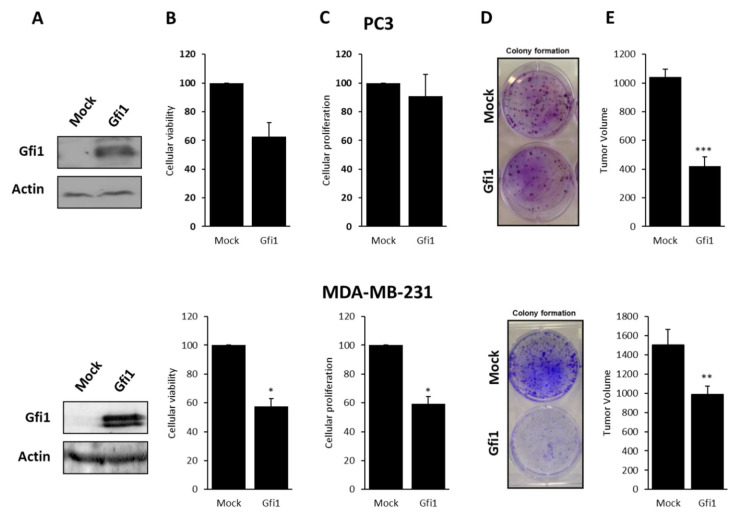
Tumor-suppressor-like properties of Gfi1 re-expression. (**A**) Western Blot showing Gfi1 expression in empty vector and Gfi1-transfected PC3 and MDA-MB-231 cells. (**B**) Effect of Gfi1 expression on PC3 and MDA-MB-231 cellular viability monitored by MTT assay. (**C**) Cell proliferation of empty vector and Gfi1-transfected PC3 and MDA-MB-231 cells. (**D**) Colony formation assay of PC3 and MDA-MB-231 cells transfected with the empty vector or with Gfi1. (**E**) Effect of Gfi1 transfection on the in vivo growth of PC3 and MDA-MB-231 cells. Tumor size was monitored over time, and size is shown in cubic millimeters. * *p* < 0.05, ** *p* < 0.01, *** *p* < 0.001.

Tumor suppressor properties have been proposed for Gfi1 since its re-expression in cancer cell lines lacking Gfi1 function decreases cell proliferation [16,20]. We assessed the ability of Gfi1 to function as tumor suppressor gene in prostate and breast cancer cell lines. PC3 and MDA-MB-231 cell lines were stably transfected with Gfi1 and clones expressing high Gfi1 levels were selected (Figure 3). The effect of Gfi1 on cell viability and proliferation was assessed by MTT assay and the incorporation of ^3^H-timidine respectively in the transfected cell lines. Gfi1 re-expression decreased cell viability to 57% and cell proliferation rate was reduced to 59% in MDA-MB-231. In PC3 cells, Gfi1 expression reduced cell viability but there were no significant changes in the incorporation of ^3^H-timidine experiments. In addition, both cells lines showed a significantly reduced percentage of colony formation density when expressing Gfi1 (Figure 3).

Then, we moved to the in vivo experiments by analyzing the ability of Gfi1-transfected MDA-MB-231 and PC3 cells to form tumors in nude mice compared to the empty vector-transfected cells. The same mice were injected in both flanks with 10^7^ MDA-MB-231 or PC3 cells transfected with Gfi1 or empty vector, and tumor growth was monitored every 3 days. As shown in Figure 3, the tumors generated by Gfi1 transfected cells grow slower than the tumors from empty vector-transfected cells. Finally, the effects of Gfi1 re-expression on cell migration and invasion were assessed, but no significant changes were found (Supplementary Figure S1).

**Figure 4:**
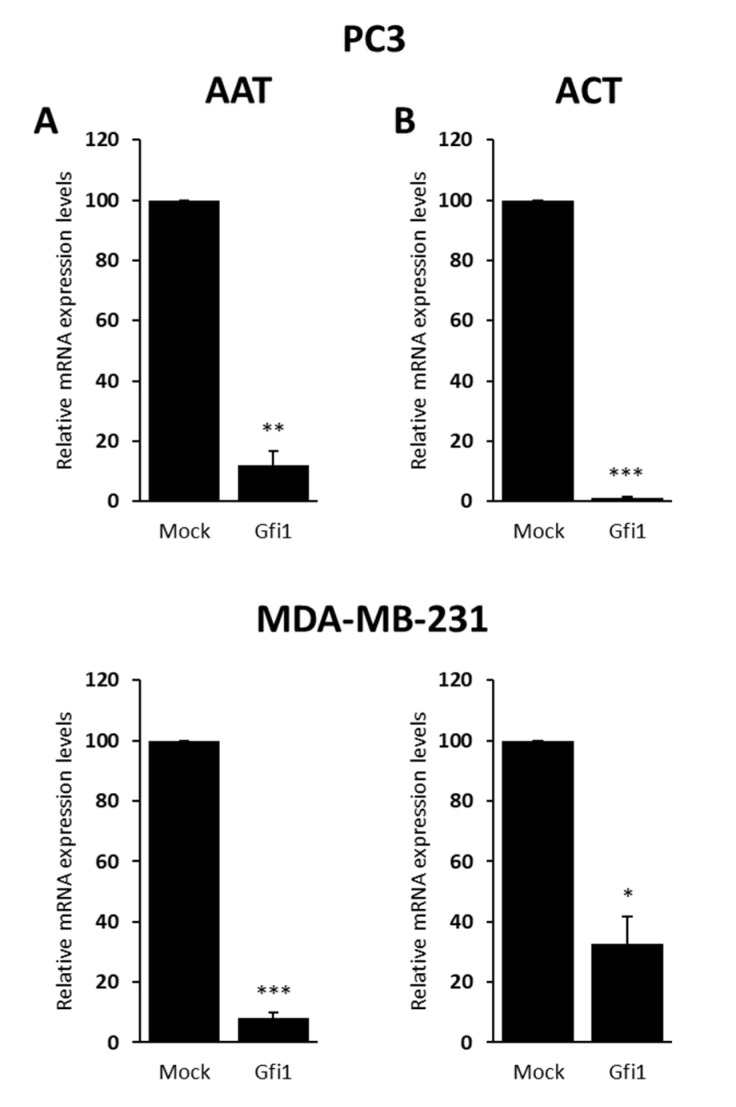
Analysis of expression of Gfi1 target genes in PC3 and MDA-MB-231 cell lines. QRT-PCR showing the mRNA expression levels of AAT (**A**) and ACT (**B**) in empty vector and Gfi1-transfected PC3 and MDA-MB-231 cells. Gfi1 re-expression reduces AAT and ACT in both cell lines. Data shown represent the mean ± s.e.m. of three independent experiments completed in triplicate. * *p* < 0.05, ** *p* < 0.01, *** *p* < 0.001

Together, these data indicate that Gfi1 inhibits tumor cell growth. Since this gene codifies for a zinc finger transcription factor, it should exert its effects on tumor development by regulating gene expression. We thus analyzed the effect of Gfi1 re-introduction on the expression of Gfi1 targets [21] with known functions in prostate and breast. In particular, mRNA levels of the alpha 1-anti-trypsin (AAT), alpha 1-anti-chymotrypsin (ACT), the transcription factor CAAT/enhancer-binding protein (C/EBP), the cell cycle regulators E2F5, Ets2, c-myc, and Neurog3 were assessed by QRT-PCR. Gfi1 re-introduction had no effect on the expression levels of CAAT/enhancer-binding protein (C/EBP), E2F5, Ets2, c-myc, and slightly decreased Neurog3 in MDA-MB-231 cells (data not shown), but significantly reduced AAT (Figure 4A), and ACT (Figure 4B) in PC3 and MDA-MB-231 cell lines transfected with Gfi1 when compared with the control cells transfected with the empty vector.

### 2.3. Gfi1 Methylation and Expression in Primary Tumors

**Figure 5:**
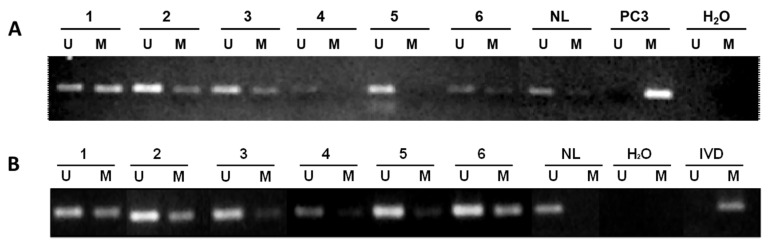
Analysis of Gfi1 methylation in primary human tumors. (**A**) Prostate cancer. (**B**) Breast cancer. The presence of a PCR band under the lane M indicates methylated genes, whereas the presence of a PCR band under the lane U indicates unmethylated genes. Normal lymphocytes (NL) and in vitro methylated DNA (IVD) and DNA from PC3 cells are used as negative and positive control for unmethylated and methylated genes, respectively.

Once the epigenetic loss of Gfi1 function on prostate and breast cancer cell lines was demonstrated, the prevalence of Gfi1 methylation in primary tumors was determined by MSP. As shown in Figure 5, Gfi1 was methylated in 51% of prostate tumor samples (*n* = 39) recapitulating our previous findings [18], and in 40% of the primary breast tumors analyzed (*n* = 44). Although we have not analyzed Gfi1 methylation prevalence in other tumor types, pooled data from cell lines and primary tumors strongly indicate that Gfi1 hypermethylation is a tumor-specific event.

**Figure 6:**
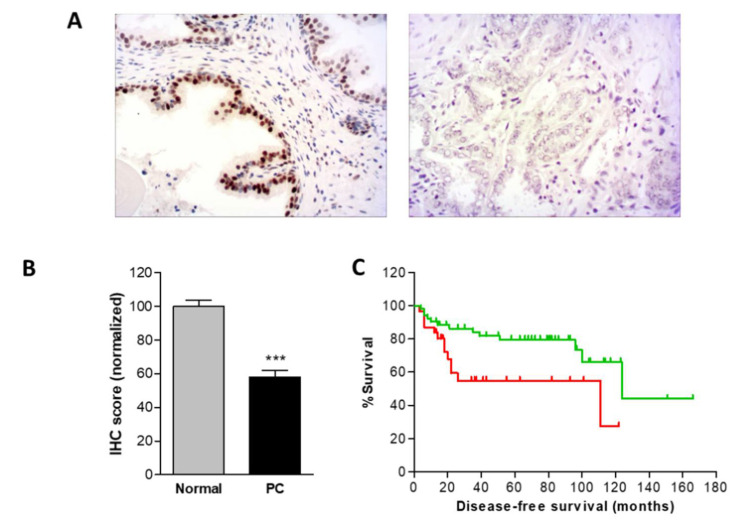
(**A**) Micrographs from normal glands (N) and prostate cancers (PT) (200×). Immunolocalization of Gfi1 (brown staining). The signals for Gfi1 in normal glands were intense. In turn, most of the tumor cells showed an evident loss of the signal for Gfi1. (**B**) Comparison of Gfi1 expression in normal (normal) and tumoral prostate tissues (PC) measured by immunohistochemistry. Gfi1 expression was significantly reduced in tumor samples. (**C**) Disease specific survival of patients showing methylated (red line) or unmethylated (green line) Gfi1. *** *p* < 0.001

In addition, the data from the immunohistochemistry experiments showed a significant decrease of Gfi1 expression in prostate cancer tissues respect normal prostate and normal-adjacent tissue suggesting a direct link between Gfi1 methylation and expression (Figure 6A). Moreover, no differences were found between normal and normal-adjacent tissues. Finally, analyzing clinical follow yielded Gfi1 methylation implied a worse disease-free survival of prostate cancer patients (log-rank, *p =* 0.0161) (Figure 6B), suggesting Gfi1 epigenetic silencing as a promising biomarker for prostate cancer progression. In addition, Gfi1 was more frequently methylated in prostate cancer samples with Gleason > 8 (48%) than in samples with Gleason ≤ 7 (22%) (*p* = 0.0213).

## 3. Discussion

In this work, we have found that Gfi1 silencing by promoter hypermethylation is a common event in prostate and breast cancer. We also showed that Gfi1 could have an important role regulating important cellular processes such as cell proliferation and tumor growth in these tumor types. Until now, most of the studies have focused on the importance of Gfi1 function in the hematopoietic system. The pioneer studies rely on its role in lymphoma development since it was originally found as a common target for insertional mutagenesis in T cell lymphomas [22]. Gfi1 is frequently mutated in patients with severe congenital neutropenia (SCN) [23], leukemia [12], and lymphoma [22], suggesting a role as an oncogene in this tissue. However, the available data are contradictory. Gfi1 inhibits hematopoietic stem cell proliferation and is essential to preserving its functional integrity [5]. In addition, Gfi1 inhibits proliferation and colony formation of p210BCR/ABL-expressing cells via transcriptional repression of STAT5 and Mcl-1 [24]. A similar scenario is found in solid tissues such as lung, inner ear hair epithelium, nervous system, and intestinal epithelium, in which Gfi1 function depends on the tissue, again suggesting a dual role for this transcription factor [16]. Two recent studies reported Gfi1 downregulation in colorectal cancer suggesting a tumor suppressor role in this tissue [17,20]. Our data can be explained by proposing a similar role for Gfi1 in prostate and breast cancer. Supporting this idea, we found that Gfi1 re-expression reduces cell viability, inhibits cell proliferation, colony formation, and tumor growth in mice xenografts of cancer cell lines with loss of Gfi1 expression.

Gfi1 has been extensively characterized as a transcriptional repressor that exerts its function by recruiting multiprotein complexes to gene promoters that, among others, include epigenetic modifier proteins. Those protein complexes also include other transcription factors that have been proposed as putative tumor suppressor genes. Gfi1 interacts with the transcriptional repressor PRDM5 to regulate gene transcription [25]. PRDM5 has been proposed as a tumor suppressor frequently silenced in multiple human tumors that antagonizes WNT/β−catenin signaling and oncogene expression [26]. Gfi1 also interacts with CBFA2T3, a transcriptional repressor that belongs to the ETO family of proteins and has been proposed as a putative breast tumor suppressor gene [27]. All these data point to Gfi1 as a potential tumor suppressor in prostate and breast cancer.

Gfi1 regulates a variety of functions such as cell differentiation, cell proliferation, cell cycle, and apoptosis by modulating the expression of a wide range of genes that includes cell-surface receptors, regulators of cell cycle, transcription factors, and cytokines among others. Here we found that Gfi1 repress AAT and ACT expression in prostate and breast cancer cell lines, while did not change the expression of genes with specific functions in the hematopoietic system. Thus, in our systems Gfi1 silencing by hypermethylation increase AAT and ACT expression in concordance with the studies showing their association with tumorigenicity of various tumor types [28,29]. AAT and ACT are serine proteinase inhibitors produced by various tumor cells. Several works found elevated AAT levels in serum in the course of a large number of malignant diseases and different cancers, including prostate and breast cancer; although these levels correlate with cancer stage and aggressiveness, its role in cancer biology is unclear [30,31]. In this way, AAT down regulates tumor necrosis factor-induced apoptosis, suggesting that elevated AAT levels may, therefore, promote cell survival and tumor growth. In breast cancer cells AAT can induce proliferation, invasiveness, and nuclear factor kappa B (NF-κB) activity [32]. Although ACT biological function is not yet fully understood, it has been suggested that local synthesis of ACT, may be implicated in the regulation of the tumorigenic potential of breast cancer cells, suggesting a role of ACT in breast cancer progression [33]. Although there is no data about the biological function of ACT in prostate cancer, it has been described that ACT stabilizes PSA plasma levels and the complex ACT-PSA is higher than PSA alone in serum from prostate cancer [34]. Additional functional studies are needed to determine the exact role of AAT and ACT in prostate and breast cancer.

Our data also showed that Gfi1 methylation implied a worse disease-free survival of prostate cancer patients suggesting Gfi1 epigenetic silencing as a promising biomarker for prostate cancer progression. Prediction of prognosis is another challenge in prostate cancer research. The Gleason score is currently the best-known risk stratification tool [35]. However, the interobserver variability found makes it necessary to establish new markers for prostate cancer prognosis. Here, we found Gfi1 more frequently methylated in samples with a high Gleason score. Moreover, in a previous study, we found that Gfi1 methylation predicted tumor progression despite androgen deprivation [36]. Although more studies with a larger number of samples may be necessary, our data indicated that Gfi1 methylation could be a good biomarker for prostate cancer progression.

In summary, our results show for the first time the epigenetic silencing of Gfi1 in prostate and breast cancer, and that this event could be a crucial step in the development of these two-well characterized endocrine-related tumors. Moreover, Gfi1 methylation is associated with worse disease-specific survival. Further studies must be done to elucidate the exact role of Gfi1 in these tumor types and their clinical implications.

## 4. Materials and Methods 

### 4.1. Cell Lines and Tumor Samples

LNCaP, PC3, and DU145 human prostate cancer cells and the human breast cancer cells MDA-MB-231, and BT-474 were obtained from the American Type Culture Collection (Manassaa, VA, USA) and routinely cultured in the appropriate medium. Cells were maintained at 37 °C in a humidified atmosphere of 95% air and 5% CO_2_.

For the treatment with the demethylating agent 5’-aza-2’ deoxycytidine (Sigma-Aldrich, St Louis, MO, USA), LNCaP, PC3, DU145, MDA-MB-231 and BT-474 cells were treated with 2 or 5 µM (Sigma-Aldrich, St Louis, MO, USA) for 3 days. Treatment was refreshed every 24 h.

Gfi1 methylation analysis were retrospectively evaluated in 44 breast tumor samples and 39 prostate tumor samples from paraffin-embedded blocks. Gfi1 expression was evaluated in 91 prostate tumor samples and 10 prostate normal tissues. Disease-free survival analysis was performed with same cohort in a previous study [17]. Clinical evolution based on PSA and imaging was recorded. The primary endpoint assessed was disease-specific survival. The study was approved by the Ethics Committee of Hospital Universitario de Getafe (A17-11 of 27 October 2011).

### 4.2. DNA Extraction

Genomic DNA isolation from cell lines and paraffin-embedded blocks was performed according to a standard phenol/chloroform/isoamyl alcohol extraction protocol, after a proteinase K digestion.

### 4.3. DNA Methylation Analysis

We established Gfi1 island methylation status by PCR analysis of bisulfite-modified genomic DNA, which induces chemical conversion of unmethylated, but not methylated, cytosine to uracil, using two procedures. First, methylation status was analyzed by bisulfite genomic sequencing of both strands of the corresponding CpG island. Primers were designed using the Methyl Primer Express v1.0 software (Applied Biosystems, Foster city, CA, USA). PCR products were loaded onto 1.5% agarose gels, stained with ethidium bromide and visualized under UV transilluminator. DNA was extracted using QIAquick Gel Extractin Kit and ligated into pGEM-T easy plasmid (Promega, Madison, WI, USA). The plasmid was transformed in competent bacterial cells and plated onto LB/ampicillin/IPTG/X-Gal plates. A minimum of five white colonies of each sequence and sample were processed by miniprep and plasmids were sequenced automatically to determinate their methylation degree. The second analysis used methylation-specific PCR using primers specific for either the methylated or the modified unmethylated DNA. Primers were designed using the Methyl Primer Express v1.0 software (Applied Biosystems, Foster city, CA, USA). Primer sequences and annealing temperatures used are available upon request. DNA from normal lymphocytes treated in vitro with SssI methyltransferase was used as a positive control for methylated alleles. DNA from normal lymphocytes was used as a positive control for unmethylated alleles. PCR products were loaded onto 1.5% agarose gels, stained with ethidium bromide and visualized under UV transilluminator.

### 4.4. mRNA and Protein Analysis

Total RNA was isolated with TRIzol Reagent (Invitrogen, Carlsbad, CA, USA). 4 µg of total RNA was reverse transcribed using SuperScript III Reverse Transcriptase (Invitrogen, Carlsbad, CA, USA), and using Oligo-dT as primer. PCR amplifications were performed in 96-well optical plates in a volume of 20 µL. We used 0.2 µg of cDNA, 5 pmol of each primer, and 10 µL of 2× SYBRgreen PCR Master Mix (Applied Biosystems, Foster city, CA, USA). Primers were designed between different exons and encompassing large introns to avoid any amplification of genomic DNA. Primer sequences and annealing temperatures used are available upon request. Expression values were normalized against the expression of glyceraldehyde-3-phosphate-dehydrogenase (*GAPDH*) and used as an endogenous control to ensure cDNA quality and loading accuracy, following the ΔΔCt method. QRT-PCR was performed on an ABI 7500 Fast (Applied biosystems, Foster city, CA, USA). 

Cell lysates for protein analysis were solubilized in RIPA buffer and resolved through 10% sodium dodecyl sulfate polyacrylamide gels, transferred to a nitrocellulose membrane, and immunoblotted with a polyclonal rabbit anti-Gfi1 (Santa Cruz Biotechnology, Dallas, TX, USA). Equal loading was tested by reprobing with a polyclonal antibody against human α-tubuline (Sigma-Aldrich, St Louis, MO, USA). Immunoreactive proteins were visualized by the ECL immunodetection system (Amersham Pharmacia Biotech, Piscataway, NJ, USA) with horseradish peroxidase-conjugated secondary antibodies. Immunohistochemistry was performed on FFPE tissue using a polyclonal rabbit anti-Gfi1 at 1:50 dilution, and stained sections were evaluated by an expert pathologist in a blinded manner. 

### 4.5. Gfi1 Transfection

The Gfi1 expression vector pCMVtag3B-Gfi1 was constructed by cloning the cDNA corresponding to the Gfi1 gene from MRC5 cell line into the pCMVtag3B vector and confirmed by sequencing. For transfection experiments, we used the pCMVtag3B vector containing the Gfi1 gene or pCMVtag3B vector (Mock). Transfection was performed by electroporation 10^7^ cells in 0.8 mL PBS with 40 µg of the vector at 250 V and 975 F. After electroporation, cells were washed with PBS and seeded in fresh medium containing 20% FBS. Transfected cells were selected by the addition of G418 (600 µg/mL).

### 4.6. Cell Viability, Cell Proliferation, Colony Formation Assay and Mouse Xenograft Model

Cell viability was determined by the 3-(4,5-Dimethyl-2-yl)-2,5-ditetrazolium bromide (MTT) (Sigma-Aldrich, St Louis, MO, USA) assay. MDA-MB-231 and PC3 transfected with Gfi1 and with the empty vector were seeded onto 6-well plate and cell viability was measured for 15 days by staining with MTT and measuring the absorbance at 595 nm. Optical density was directly proportional to cell number up to the maximum density measured.

Cell proliferation was measured by DNA synthesis assay. Cells were starved for 48 h and cell growth was induced for 12 h by the addition of whole media. Then cells were pulsed with 1 mL [^3^H]-thymidine (0.4 mCi/mL) (Amersham, GE Healthcare, Buckinghamshire, UK) for 4 h at 37 °C. After washing three times with PBS, cells were incubated with 1 mL ice-cold 5% TCA for 15 min at 4 °C, washed three times with absolute methanol, airdried and the TCA-precipitable fraction was solubilized in 500 mL of 0.1 M NaOH–1% SDS. [^3^H]-thymidine incorporation was determined with a scintillation counter (Wallac, Turku, Finland)

Colony formation assay was performed by seeding 1000 cells onto six-well plates and maintaining them on selection media. After 14 days, cells were stained and fixed and colonies were quantified. 

To measure in vivo cell proliferation, six-week-old male athymic nude mice nu/nu were used for MDA-MB-MD 231 and PC3 tumor xenograft. Seven specimens were used. Both flanks of each animal were injected with 6 × 10^6^ cells in a total volume of 50 µL of PBS. The right flank was used for Gfi1-MDA-MB-231 or PC3 transfected cells and the left for empty vector MDA-MB-231 or PC3 control cells. Tumor development at the site of injection was measured two times per week. Animals were sacrificed at 37 days. 

## Supplementary Materials

The following are available online at https://www.mdpi.com/1422-0067/21/13/4687/s1, Figure S1: Cellular migration and invasion of empty vector and Gfi1- transfected MDA-MB-231 and PC3 cells. Cells that migrated through the insert were counted after staining with crystal violet.

## References

[1] Maximov P.Y., Abderrahman B., Curpan R.F., Hawsawi Y.M., Fan P., Jordan V.C. (2018). A unifying biology of sex steroid-induced apoptosis in prostate and breast cancers. Endocr. Relat. Cancer.

[2] ZweidlerMcKay P.A., Grimes H.L., Flubacher M.M., Tsichlis P.N. (1996). Gfi-1 encodes a nuclear zinc finger protein that binds DNA and functions as a transcriptional repressor. Mol. Cell Biol..

[3] Yucel R., Karsunky H., Klein-Hitpass L., Moroy T. (2003). The transcriptional repressor Gfi1 affects development of early, uncommitted c-Kit+ T cell progenitors and CD4/CD8 lineage decision in the thymus. J. Exp. Med..

[4] Person R.E., Li F.Q., Duan Z., Benson K.F., Wechsler J., Papadaki H.A., Eliopoulos G., Kaufman C., Bertolone S.J., Nakamoto B. (2003). Mutations in proto-oncogene GFI1 cause human neutropenia and target ELA2. Nat. Genet..

[5] Hock H., Hamblen M.J., Rooke H.M., Schindler J.W., Saleque S., Fujiwara Y., Orkin S.H. (2004). Gfi-1 restricts proliferation and preserves functional integrity of haematopoietic stem cells. Nature.

[6] Zeng H., Yucel R., Kosan C., Klein-Hitpass L., Moroy T. (2004). Transcription factor Gfi1 regulates self-renewal and engraftment of hematopoietic stem cells. EMBO J..

[7] Wallis D., Hamblen M., Zhou Y., Venken K.J.T., Schumacher A., Grimes H.L., Zoghbi H.Y., Orkin S.H., Bellen H.J. (2003). The zinc finger transcription factor Gfi1, implicated in lymphomagenesis, is required for inner ear hair cell differentiation and survival. Development.

[8] Tsuda H., Jafar-Nejad H., Patel A.J., Sun Y., Chen H.K., Rose M.F., Venken K.J.T., Botas J., Orr H.T., Bellen H.J. (2005). The AXH domain of Ataxin-1 mediates neurodegeneration through its interaction with Gfi-1/senseless proteins. Cell.

[9] Kazanjian A., Wallis D., Au N., Nigam R., Venken K.J.T., Cagle P.T., Dickey B.F., Bellen H.J., Gilks C.B., Grimes H.L. (2004). Growth factor independence-1 is expressed in primary human neuroendocrine lung carcinomas and mediates the differentiation of murine pulmonary neuroendocrine cells. Cancer Res..

[10] Shroyer N.F., Wallis D., Venken K.J., Bellen H.J., Zoghbi H.Y. (2005). Gfi1 functions downstream of Math1 to control intestinal secretory cell subtype allocation and differentiation. Genes Dev..

[11] Bjerknes M., Cheng H. (2010). Cell Lineage metastability in Gfi1-deficient mouse intestinal epithelium. Dev. Biol..

[12] Khandanpour C., Thiede C., Valk P.J.M., Sharif-Askari E., Nuckel H., Lohmann D., Horsthemke B., Siffert W., Neubauer A., Grzeschik K.H. (2010). A variant allele of Growth Factor Independence 1 (GFI1) is associated with acute myeloid leukemia. Blood.

[13] Vassen L., Khandanpour C., Ebeling P., van der Reijden B.A., Jansen J.H., Mahlmann S., Duhrsen U., Moroy T. (2009). Growth factor independent 1b (Gfi1b) and a new splice variant of Gfi1b are highly expressed in patients with acute and chronic leukemia. Int. J. Hematol..

[14] Doan L.L., Kitay M.K., Yu Q., Singer A., Herblot S., Hoang T., Bear S.E., Morse H.C., Tsichlis P.N., Grimes H.L. (2003). Growth factor independence-1B expression leads to defects in T cell activation, IL-7 receptor alpha expression, and T cell lineage commitment. J. Immunol..

[15] Schmidt T., Karsunky H., Gau E., Zevnik B., Elsasser H.P., Moroy T. (1998). Zinc finger protein GFI-1 has low oncogenic potential but cooperates strongly with pim and myc genes in T-cell lymphomagenesis. Oncogene.

[16] Kazanjian A., Gross E.A., Grimes H.L. (2006). The growth factor independence-1 transcription factor: New functions and new insights. Crit. Rev. Oncol. Hematol..

[17] Xing W., Xiao Y., Lu X., Zhu H., He X., Huang W., Lopez E.S., Wong J., Ju H., Tian L. (2017). GFI1 downregulation promotes inflammation-linked metastasis of colorectal cancer. Cell Death Differ..

[18] Ashour N., Angulo J.C., Andres G., Alelu R., Gonzalez-Corpas A., Toledo M.V., Rodriguez-Barbero J.M., Lopez J.I., Sanchez-Chapado M., Ropero S. (2014). A DNA hypermethylation profile reveals new potential biomarkers for prostate cancer diagnosis and prognosis. Prostate.

[19] Rau K.M., Kang H.Y., Cha T.L., Miller S.A., Hung M.C. (2005). The mechanisms and managements of hormone-therapy resistance in breast and prostate cancers. Endocr. Relat. Cancer.

[20] Chen M.S., Lo Y.H., Chen X., Williams C.S., Donnelly J.M., Criss Z.K., Patel S., Butkus J.M., Dubrulle J., Finegold M.J. (2019). *Growth Factor-Independent 1* Is a Tumor Suppressor Gene in Colorectal Cancer. Mol. Cancer Res..

[21] Duan Z., Horwitz M. (2003). Targets of the transcriptional repressor oncoprotein Gfi-1. Proc. Natl. Acad. Sci. USA.

[22] Gilks C.B., Bear S.E., Grimes H.L., Tsichlis P.N. (1993). Progression of interleukin-2 (IL-2)-dependent rat T cell lymphoma lines to IL-2-independent growth following activation of a gene (Gfi-1) encoding a novel zinc finger protein. Mol. Cell Biol..

[23] Boztug K., Klein C. (2009). Novel genetic etiologies of severe congenital neutropenia. Curr. Opin. Immunol..

[24] Soliera A.R., Mariani S.A., Audia A., Lidonnici M.R., Addya S., Ferrari-Amorotti G., Cattelani S., Manzotti G., Fragliasso V., Peterson L. (2012). Gfi-1 inhibits proliferation and colony formation of p210BCR/ABL-expressing cells via transcriptional repression of STAT 5 and Mcl-1. Leukemia.

[25] Duan Z., Person R.E., Lee H.H., Huang S., Donadieu J., Badolato R., Grimes H.L., Papayannopoulou T., Horwitz M.S. (2007). Epigenetic regulation of protein-coding and microRNA genes by the Gfi1-interacting tumor suppressor PRDM5. Mol. Cell Biol..

[26] Shu X.S., Geng H., Li L.L., Ying J.M., Ma C.H., Wang Y.J., Poon F.F., Wang X., Ying Y., Yeo W. (2011). The Epigenetic Modifier PRDM5 Functions as a Tumor Suppressor through Modulating WNT/beta-Catenin Signaling and Is Frequently Silenced in Multiple Tumors. PLoS ONE.

[27] Kumar R., Manning J., Spendlove H.E., Kremmidiotis G., McKirdy R., Lee J., Millband D.N., Cheney K.M., Stampfer M.R., Dwivedi P.P. (2006). ZNF652, a novel zinc finger protein, interacts with the putative breast tumor suppressor CBFA2T3 to repress transcription. Mol. Cancer Res..

[28] Chawla R.K., Lawson D.H., Sarma P.R., Nixon D.W., Travis J. (1987). Serum alpha-1 proteinase inhibitor in advanced cancer: Mass variants and functionally inert forms. Cancer Res..

[29] Tountas Y., Sparos L., Theodoropoulos C., Trichopoulos D. (1985). Alpha 1-antitrypsin and cancer of the pancreas. Digestion.

[30] El-Akawi Z.J., Al-Hindawi F.K., Bashir N.A. (2008). Alpha-1 antitrypsin (alpha1-AT) plasma levels in lung, prostate and breast cancer patients. Neuro Endocrinol. Lett..

[31] El-Akawi Z.J., Abu-Awad A.M., Sharara A.M., Khader Y. (2010). The importance of alpha-1 antitrypsin (alpha1-AT) and neopterin serum levels in the evaluation of non-small cell lung and prostate cancer patients. Neuro Endocrinol. Lett..

[32] Zelvyte I., Lindgren S., Janciauskiene S. (2003). Multiple effects of alpha1-antitrypsin on breast carcinoma MDA-MB 468 cell growth and invasiveness. Eur. J. Cancer Prev..

[33] Finlay T.H., Tamir S., Kadner S.S., Cruz M.R., Yavelow J., Levitz M. (1993). alpha 1-Antitrypsin- and anchorage-independent growth of MCF-7 breast cancer cells. Endocrinology.

[34] Brawer M.K., Ferreri L.F., Bankson D.D. (2000). Long-term stability of alpha-1-antichymotrypsin complexed form of prostate specific antigen. Prostate Cancer Prostatic Dis..

[35] Epstein J.I., Egevad L., Amin M.B., Delahunt B., Srigley J.R., Humphrey P.A., Grading Committee (2016). The 2014 International Society of Urological Pathology (ISUP) Consensus Conference on Gleason Grading of Prostatic Carcinoma: Definition of Grading Patterns and Proposal for a New Grading System. Am. J. Sur. Pathol..

[36] Angulo J.C., Andrés G., Ashour N., Sánchez-Chapado M., López J.I., Ropero S. (2016). Development of Castration Resistant Prostate Cancer can be Predicted by a DNA Hypermethylation Profile. J. Urol..

