# Supplementary material for: Epigenetic Regulation of Gfi1 in Endocrine-Related Cancers: A Role Regulating Tumor Growth
Source: Int J Mol Sci. 2020 Jun 30;21(13):4687. doi: 10.3390/ijms21134687 (PMC7370116; doi:10.3390/ijms21134687)
Supplement: Supplementary file 1 [file ijms-21-04687-s001.pdf]

Supplementary information

Supplementary methods

Migration and invasion Assays

Migration assays were performed in transwell insert with 8 μm pore uncoated membrane filters (Corning Incorpored). Transwell were covered with 0.1 μg/μL of fibronectin (Sigma-Aldrich). Transfected cells were trypsinized, resuspended in serum-free DMEN or RPMI and transferred to the upper chamber. 600 μl of growth medium containing 10% FBS was added to the bottom wells. The cells were cultured at 37°C and 5% CO_2 for 24 hours. Following incubation, the medium was aspired, and the cells remaining on the upper surface of the filter were stained with crystal violet for 1 hour. The average number of migrated cell were determined by counting the cells in 3 random high power field (10x).

Invasion assays were performed in matrigel chambers (BD). Matrigels invasion chambers were rehydraded with serum-free DMEN or RPMI at 37°C and 5% CO_2 for 1 hour. Transfected cells were trysinized, resuspended in serum-free DMEN or RPMI and transferred to the upper chamber. 600 μl of growth medium containing 10% FBS was added to the bottom wells. The cell were cultured at 37°C and 5% CO_2 for 24 hours. Following incubation, the medium were aspired, and the cells remaining on the upper surface of the filter were stained with crystal violet for 1 hour. The average number of migrated cell were determined by counting the cells in 3 random high power field (10x).

Supplementary Figure 1

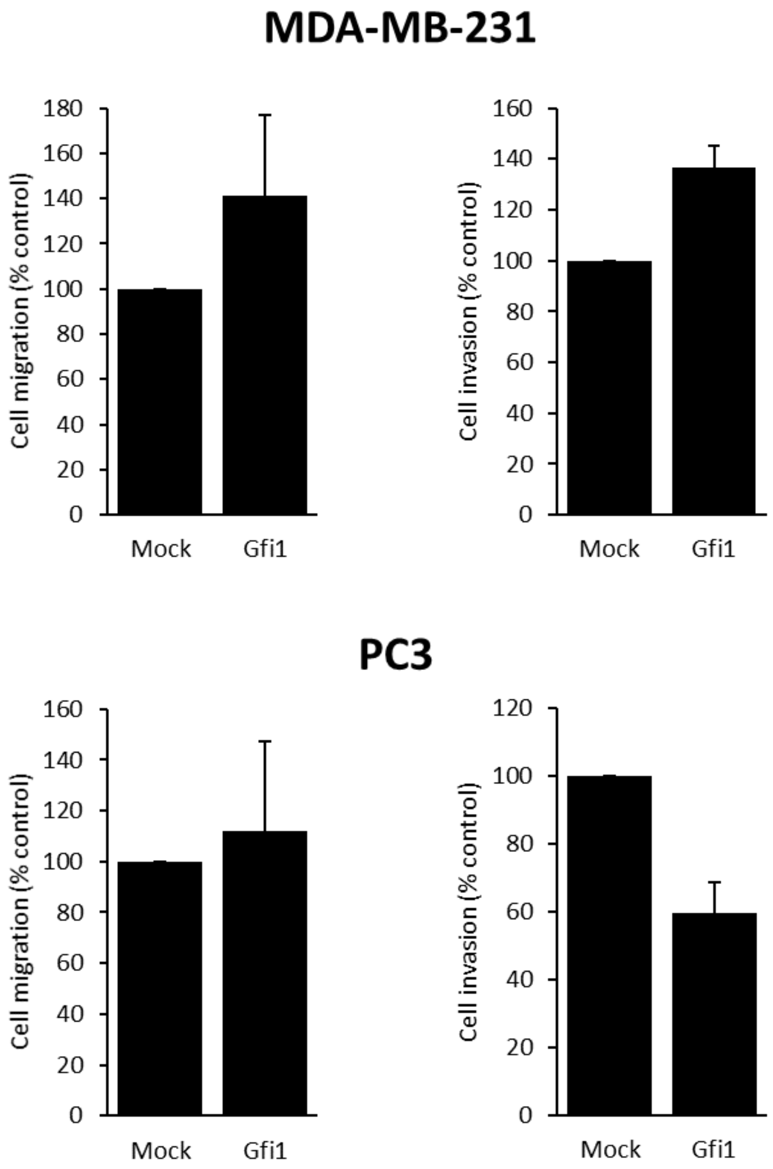

Supplementary figure 1. Cellular migration and invasion of empty vector and Gfi1-transfected MDA-MB-231 and PC3 cells. Cells that migrated through the insert were counted after staining with crystal violet.
